# History of the Excision of a Portion of the Ribs and Pleura

**Published:** 1818-10

**Authors:** M. Richerand

**Affiliations:** Principal Surgeon of the Hospital St. Louis.


*For the London Medical and Physical Journal.*

### *History of the Excision of a Portion of the Ribs and Pleura;* by M. Richerand, Principal Surgeon of the Hospital St. Louis.

[From our Correspondent at Paris.]

M. MICHELLEAU, of Nemours, for the space of three years, laboured under a cancerous tumour in the region of the heart; the extirpation of which was performed

---

4

by a surgeon in the neighbourhood in the month of January. On removing the first dressing, a bloody fungus appeared in the centre of the wound, which, although cauterised daily, rapidly increased. A second operation was tried; the parts were excised more deeply. After having exposed the ribs, the surgeon cut down even to the pleura; new fungosities however appeared, and increased, notwithstanding repeated cauterization, by means of which their removal was attempted. In despair, from the want of success after such painful operations, the patient came to Paris towards the end of March, with a determination to undergo any suffering with the hope of being delivered from a horrible evil, and of escaping an apparently certain death.

At this period, an enormous fungus arose from the wound; it was brown and soft, exuding an abundant reddish sanies, so fœtid that it was impossible to remain a quarter of an hour with the patient without renewing the air of the apartment: the pains, nevertheless, were moderate; there was no colliquative perspiration or diarrhœa. In this state of things, it was determined to cut away that portion of the ribs from which the cancer originally took its birth. I did not conceal from the patient the probability that I should be obliged to remove a portion of the pleura: he did not hesitate to submit to the operation, the serious nature of which he was fully able to appreciate.

On the 31st of March, I proceeded upon this bold undertaking with the friendly assistance of my colleague, Professor Dupuytren, and by other persons who gave me their cooperation. I began by enlarging the wound, giving it a crucial form; I thus laid bare the sixth rib, which appeared swelled and *rugueuse* for four inches in length, with a blunt-pointed bistoury, whose point I conducted along its upper and lower margin; I cut the intercostal muscles; then, with a little saw, I divided the bone at the two extremities of the diseased portion, and removed it from the pleura by a spatula. This I effected with an unexpected facility, in consequence of the thickening of the pleura beneath the bone, which became manifest in the sequel of the operation.

The seventh rib was discovered in the same extent, isolated and detached in the same manner, but with much more difficulty and slight laceration. The pleura then appeared evidently diseased; a thickened fungous giving rise to the tumour which occupied the space between the two portions of the ribs which I removed. The cancerous structure prolonged itself above the sixth rib, so that the membrane appeared diseased for the space of about eight inches square of its extent. Not to remove this, would have left our operation incomplete. Each of the assistants provided himself with the means of arresting the hæmorrhage we expected in an alarming degree from the section of the intercostal arteries. I cut the pleura with curved scissars; and, whether the section performed by this instrument (which acts by pressing rather than incision) occasioned the retraction of the vessels, or whether their calibre had been diminished by antecedent cauterisations, not a drop of blood flowed. The external air at this moment rushed rapidly into the chest, and threatened immediate suffocation. This I endeavoured to diminish, by covering as speedily as possible the chest, surrounding the opening with a compress spread with cerate; over this I placed a large and thick pad of lint, and the whole was confined by a moderately tight bandage. The anxiety and difficulty of breathing were extreme during twelve hours after the operation; the patient passed the entire night sitting in an erect posture; towards the morning, sinapisms, applied to the soles of the feet and the inner part of the thighs, relieved the respiration; the pulse became stronger, and the strength of the patient increased. The only liquid or nourishment allowed was an infusion of linden flowers and violets, aromatized with orange-flower water, and sweetened with syrup of gum arabic.

Three days passed in this manner. The fever was moderate, but the oppression deprived the patient of sleep: the first dressing was removed ninety-six hours after the operation. The pericardium and lungs had contracted an adhesion with the circumference of the quadrilateral opening, *a sort of window made* in front of the heart; the adhesion happily was not complete between the pericardium and the lung from the sixth to the twelfth day; by this means, an abundant flow of serum came from the chest each dressing, probably not less than a pint (English), half-a-pint (French), in the twenty-six hours. On the thirteenth day, this serosity, produced by the inflammation of the surfaces, ceased to flow; and, on the eighteenth, the adhesion was complete between the lung and pericardium; the air immediately ceased to enter into the wound; the patient could lie on his side; the appetite and sleep were entirely re-established. The wound diminished rapidly, and presented a most favourable appearance; on the twenty-seventh day after the operation, the patient returned home, with directions to have the part well protected when the cicatrix was completed.

I did not suffer the opportunity to escape me of demonstrating anew the perfect insensibility of the heart and pericardium: the contact of the fingers to these organs was not perceived by the patient. The pericardium of the human being during life is so transparent, that the heart is as readily distinguished as if it were under a perfectly diaphanous glass; so much so, that we sometimes thought the envelop was wanting. This transparence is wanting in the dead body; and, in this respect, may be compared to the cornea of the eye, which becomes obscured and opake at the approach of death.

A large opening, with loss of substance, made in the parietes of the chest, not being necessarily followed by suffocation, or by dangerous inflammation of the thoracic viscera, we might, in a case where the individual labours under dropsy of the pericardium, make an opening in the front of the heart; an opening which would permit not only the evacuation of the water, but radically to cure the disease by the production of adhesive inflammation of the surfaces, by means similar to those employed for the cure of hydrocele. This may appear a rash enterprize; but how many operations, reputed impossible fifty years back, are now followed by the most brilliant success.

---

*On the Use of some of the Salts of Morphine.* By M. Majendie. (From the same.)

A young lady, æt. 24, was attacked for the space of ten years, with a disease which I considered an aneurism of the pectoral aorta; had taken the opinion of a variety of physicians without effect. She was tormented by continual wakefulness; pains extremely acute in the region of the diaphragm, and in the lower limbs, which were in part wasted.

I first employed the prussic acid with advantage; but I was obliged to cease after about six weeks, because it occasioned painful and fatiguing dreams. I then determined to try the salts of morphine, which, from experiments on dogs, I had found to be powerful narcotics. I ordered four pills, each containing a quarter of a grain of *acetate of morphine*, and directed one to be taken at night going to bed, and a second in the morning: at night, she took a pill, which not producing the desired effect, in half an hour she took a second, after which she slept profoundly, which she had not experienced for many months; in the night she awoke after three or four hours' quiet sleep, was sick, but slept again; this happened several times: at six o'clock, she made some efforts to vomit, and rejected a small quantity of mucus and bile; but she remained in a state of freedom from pain she had not before experienced. It was evident the dose had been carried too far; I recommended her to take two pills in the twenty-four hours, each containing only the eighth of a grain, which she employed for six months, and always with advantage. It is somewhat remarkable, that, after this long continuance, the effect of the remedy is not materially weakened, since she cannot exceed four in twenty-four hours without experiencing violent head-ache or nausea.

I tried upon this same patient to substitute the *muriate of morphine* for the *acetate*, but I found it necessary to employ a grain and a half; and even then the effect was less satisfactory; so that the patient would not continue its use.

The *sulphate of morphine*, tried on the same person, had a weaker action than the acetate, but much stronger than the muriate; its narcotic power is also more complete. Sleep, which it procures, is exempt from dreams; its effects are similar to the acetate, but it is less energetic.

For four months the patient has used both the sulphate and the acetate according to circumstances. The latter she calls the strong, and the former the weak pills; both contain the eighth of a grain. Sometimes she takes the one and sometimes the other, according to the degree of pain she feels.

In the course of her complaint, she desired to change the remedy from a feeling which frequently influences persons suffering under tedious chronic complaints. I gave her Derosne's essential salt, without indicating that it was an opiate: half a grain, which she took in the twenty-four hours, caused an extreme agitation and a most intense head-ache; she then returned to her salt of morphine, which she takes to this moment.

I have employed the same remedy, on other occasions, with marked advantage. A lady, labouring under a schirrus of the right breast, took, for the last two months, a quarter of a grain of acetate of morphine daily, without any other remedy; the pains, which before were lancenating, very acute, and frequent, are in a great degree calmed, and only appear at very long intervals.

---

*For the London Medical and Physical Journal.*

