# Errata

**Published:** 1818-12

**Authors:** 


— 273, — 46, for *inches square*, read *square inches*.

